# The Impact of Urban Development Intensity on Ecological Carrying Capacity: A Case Study of Ecologically Fragile Areas

**DOI:** 10.3390/ijerph18137094

**Published:** 2021-07-02

**Authors:** Jinjing Hu, Yong Huang, Jie Du

**Affiliations:** 1 School of Architecture and Urban Planning, Chongqing University, Chongqing 400045, China; joyameteor@163.com; 2 Key Laboratory of New Technology for Construction of Cities in Mountain Area (Chongqing University), Ministry of Education, Chongqing 400045, China; 3 School of Microelectronics and Communication Engineering, Chongqing University, Chongqing 400045, China; dujie@cqu.edu.cn

**Keywords:** ecologically fragile area, ecological carrying capacity, urban development intensity, the Three Gorges Reservoir Area (Chongqing section), coupling coordination degree model, geographically weighted regression model, ordinary least squares model, pressure-state-response model

## Abstract

In ecologically fragile areas, an uncontrolled increase in urban development intensity (UDI) will erode the ecological carrying capacity (ECC). This study aimed to explore the relationship between UDI and ECC and quantify the impacts of UDI on ECC. The Three Gorges Reservoir Area (Chongqing section) was chosen for the case study. Firstly, the UDI and ECC were comprehensively evaluated. Then, the coupling coordination relationship between the two was analyzed by a coupling coordination degree model. Finally, the influences of UDI on the coordinated development of the two were analyzed by a geographically weighted regression model. The results show that the distributions of UDI and ECC are opposite; UDI and ECC are mutually restricted to some extent. UDI and ECC are moderately coupled and poorly coordinated, and a higher UDI is mostly correlated to a higher coordination degree of UDI and ECC. In areas with higher UDI, an appropriate control on population and economy may benefit the coordinated development. Meanwhile, in areas with lower UDI, the promotion of population aggregation and economic investment would enhance the coordinated development between UDI and ECC. This study could optimize the dimensional control of UDI, which contributes to the long-term sustainability of ecologically fragile areas.

## 1. Introduction

According to the annual report of the China Council for international cooperation in environment and development (CCICED) (2012), more than 360 million people are living in ecologically fragile areas, accounting for about one-quarter of the total population of China [1]. Meanwhile, there is a certain overlap between ecologically fragile areas and poverty-stricken areas [2,3,4]. Therefore, equal attention to urban development in ecologically fragile areas is necessary for the sustainable development of the whole region.

Ecological fragile areas are located in the transition zone of two different types of ecosystems [5]. They are more sensitive to climate change, weak in anti-interference, and prone to various ecological problems [6,7]. Lots of cities in ecologically fragile areas are facing challenges [7,8,9]. For example, various human activities (e.g., urban construction) tend to have a huge impact on the environment, even posing a threat to ecological security [7,9]. Cities in ecologically fragile areas are in urgent need of development, which could alleviate poverty. Therefore, for cities in ecologically fragile areas, how to achieve reasonable urban development yet prevent environmental problems and ensure ecological security is an important practical question.

To address this issue, we need to investigate the impacts of urban development on the environment. Recently, many scholars have described the process of urban development from the perspectives of urbanization [10,11,12], urban expansion [13,14], land-use change [7,15], and urban forms [16], and explored the various impacts of urban development on the environment. However, these descriptions, focused on the physical changes of urban development, do not apply to the real situation of cities in ecologically fragile areas. In particular, due to the constraints of a complex terrain, the change in physical space in ecologically fragile areas is not as significant an influencing factor as the socioeconomic and demographic level [7,17]. Therefore, physical and socioeconomic explanations should be suitable for revealing the realistic changes of urban development in ecologically fragile areas.

Urban development intensity (UDI) can be defined as the multiple impacts of various human activities on urban areas [18,19,20], including land-use intensity, population density, economic intensity, etc. This term reflects the compound state of urban land use, population, and economic development in a specific period [19,20]. It can well reflect the compound changes of urban physical space and socioeconomic level, making it applicable to cities in ecologically fragile areas. Furthermore, UDI has been widely used in urban planning, land use, and other fields [18,20,21], and the control and guidance of UDI are a direct reflection of relevant planning and policy [22]. Therefore, considering UDI as the basis to reveal the relationship between urban development and the ecological environment would be beneficial for urban managers and policymakers in ecologically fragile areas.

At present, there are some studies on the impact of UDI on the environment. It is pointed out that excessive land-use intensity will reduce biodiversity [15,23,24]. Similarly, an increase in land-use intensity would reduce the ecosystem regulation services [7,14]. In addition, an increase in UDI leads to an increase in carbon dioxide emissions [16,19,25], which may aggravate the urban heat island effect. Additionally, land-use intensity is positively correlated with the PM_2.5_ concentration [26,27], which means a negative impact on air quality. These studies give some suggestions for the control and guidance of UDI. However, most of the evaluation factors for the impact selected in the above works are relatively one-sided, and attention to ecological security issues is missing. Therefore, they are not suitable for revealing the practical problems of ecologically fragile areas.

Ecological carrying capacity (ECC) [28], i.e., the environmental carrying capacity, is an important index to reflect the state of the environment. According to the theory of urban complex ecosystem [29], it is also the ability of the ecosystem to provide services, prevent ecological problems, and protect regional ecological security. Those are exactly what cities in ecologically fragile areas are concerned about. Specifically, the ECC is the comprehensive “social–economic–natural” capacity of the urban complex ecosystem [30,31,32], including support capacity, supply capacity, and coordination capacity. Therefore, it can reflect the sustainable development ability of a region and has been widely developed in urban planning, resource and environmental management, regional development, and other fields.

The evaluation methods of ECC are rich, some of which can be applied to a variety of different research scenarios [28,33,34]. In particular, the state space method, such as the pressure-state-response (PSR) model [35,36,37], can better reflect the dynamic changes of regional ECC in a certain period. This is conducive to the prevention of environmental problems caused by the weakening of ECC in advance, which is very useful for ecologically fragile areas. Thus, it is appropriate to consider the ECC assessed by the PSR model as the basis for revealing the impact of UDI on the environment in ecologically fragile areas.

The Three Gorges Reservoir Area (Chongqing section) [38,39] is located in the upper reaches of the Yangtze River, accounting for more than 85% of the whole Three Gorges Reservoir area. As an ecotone of the karst landscape ecosystem and the karst forest ecosystem in Southwest China, the Three Gorges Reservoir Area (Chongqing Section) is a typical ecologically fragile area [6,40]. Due to the complex geological conditions, the area is easily affected by various natural disasters, e.g., landslides, bank collapses, debris flows, etc. [39]. At present, research on the Three Gorges Reservoir Area (Chongqing Section) mainly focuses on the evaluation of environmental sensitivity [6], landscape change [41], and ecosystem services in the area [42], with less attention paid to an analysis of the relationship between urban development and the environment. However, urban development in the Three Gorges Reservoir Area (Chongqing section) also encounters challenges faced by other cities in ecologically fragile areas. As a case study, it has typicality and can reveal the impact of UDI on ECC.

To demonstrate the impact of UDI on ECC, it is also necessary to analyze the relationship between UDI and ECC. However, little research has been conducted on the relationship between UDI and ECC. Most research has been conducted on the relationship between urban development and the environment, which can also indirectly illustrate the relationship between UDI and ECC [10,12,43]. Meanwhile, the coupling coordination degree model has been widely used to quantify the relationship between urbanization and the environment (hereafter called relationships). For instance, relationships in the whole country [12,44], river basin [10,45], and city [44,46] have been analyzed from the macro, middle, and micro perspectives. It was found that the relationships of most regions in China was in a state of moderately coupled and weakly coordinated [12]. Subsequently, we found that there were obvious spatial differentiations in the coordination degree between urban development and the environment [12,45]. Specifically, the coordination degree in the eastern region of China with a mature urban development level was higher than that in the middle and western regions, and this trend had been constantly strengthening from 2005 to 2016 [12]. Similarly, in research from 2021 [45], it was found that the relationships of most areas in the Pearl River Delta were in a middle coupling and coordination state from 2000 to 2015. Some cities located in the relatively central parts of the Pearl River Delta, i.e., developed cities, had a higher coordination degree than the surrounding areas, forming a core-periphery spatial distribution pattern. Furthermore, we found that this phenomenon was not only affected by the spatial distribution of the cities, but also seemed to be relevant to the urban development trajectories. In 2017, a case conducted in Shanghai [46], a developed city in China, found that the coordination degree of urbanization and the environment was growing gradually from a barely coordinated stage to a highly coordinated stage from 1980 to 2013. Therefore, we believe that there is a linkage between the urban development trajectory and coordination degree growth: the higher the level of urban development, the higher the coordination degree.

These previous studies have enriched our understanding of the interaction between UDI and the ECC based on the relationship between urban development and the environment. It is mostly defined as a coupling and coordination relationship [44,45,46,47]. However, most of the studies focus on urban developed areas, while less attention is paid to developing, ecologically fragile areas. Thus, there is a lack of targeted research that can better reveal the practical problems in ecologically fragile areas. Meanwhile, it has become a common phenomenon that in areas with higher urban development, such as developed cities, the coordination degree of urbanization and the environment is higher than in other areas. However, they have, as a result, failed to provide clear and adequate evidence of what caused that common phenomenon, which is critical for further explanation of the specific impact of urban development on the environment, as well as the impact of UDI on the ECC.

To further explore the relationship between UDI and ECC in ecologically fragile areas, this paper takes the Three Gorges Reservoir Area (Chongqing section) as a typical case to explore the relationship between UDI and ECC in 2010, 2014, and 2019. First, we establish a comprehensive index system to evaluate UDI and ECC. Then, we reveal the coupling coordinated relationship between UDI and ECC by a coupling coordinated degree model. Finally, we analyze the impact of UDI on the coordination degree of UDI and ECC by a geographic weighted regression model. It is expected that the specific impact of UDI on ECC in ecologically fragile areas can be revealed to provide valuable reference suggestions for urban development strategies and management measures in ecologically fragile areas. Based on this, the urban development practice can be optimized, so that a win–win situation between high-quality urban development and environmental protection can be achieved in ecologically fragile areas.

The aims of this paper can be summarized as follows: (1) We use UDI and ECC as crucial indicators to evaluate the realistic situation of urban development and the environment in ecologically fragile areas from a dynamic perspective. (2) Motivated by this, we analyze the spatiotemporal distribution characteristics of UDI and ECC, and explore the reasons behind this phenomenon, as well as the potential relationship between UDI and ECC. (3) Then, we analyze the spatiotemporal distribution characteristics of the coupling coordination relationship between UDI and ECC, as well as the reasons behind this phenomenon. (4) Based on this, we present the in-depth influences of different realistic factors of UDI (e.g., economy, population) on the coordinated development of UDI and ECC in the ecologically fragile areas in the long term. (5) Finally, we propose some effective ways to achieve coordinated development for cities in ecologically fragile areas.

## 2. Materials and Methods

### 2.1. Study Area

**Figure 1:**
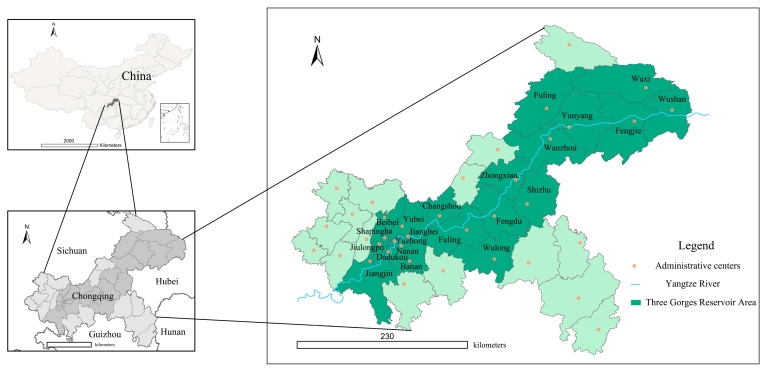
Study area. Sources: Standard Chinese Maps issued by the Ministry of Natural Resources (China) in 2020.

The Three Gorges Reservoir Area (Chongqing section) is mainly located at the end of the upper reaches of the Yangtze River [40,48]. Geographical coordinates range from 28°28′–31°44′ N and 105′N°49′–110°12′ E. It involves 22 districts and counties in Chongqing, starting from Jiangjin District in the west, Wushan County in the East, Wulong District in the south, Wuxi County, and Kaizhou District in the north, as shown in Figure 1. The whole Chongqing section covers an area of 46,158.53 km^2^ [38], accounting for 80% of the Three Gorges Reservoir Area.

According to the National Plan for the Protection of Ecologically Fragile Areas issued in 2008 [42], the Three Gorges Reservoir area (Chongqing section) is located in the southwest karst rocky desertification ecologically fragile area. It is often affected by various natural disasters, such as debris flow, soil erosion, etc. Meanwhile, the Three Gorges Reservoir area plays an important role in national ecological security. As an ecological barrier area with important ecological functions, the ecological security of this area is related to the overall security of the entire upper reaches of the Yangtze River [38].

By the end of 2009, the Three Gorges Project was completed, and the Resettlement Project in the Three Gorges Reservoir Area (Chongqing section) smoothly came to an end [42]. The Resettlement Project led to rapid urbanization in this area and caused some problems for the environment. Therefore, at the end of 2013, the government positioned the Three Gorges Reservoir Area (Chongqing section) as an Ecological Conservation and Development Zone [49]. With more emphasis on environmental protection and ecological development, this area entered the new era of urbanization transformation.

To authentically illustrate the development track of the Three Gorges Reservoir Area (Chongqing section), the years 2010, 2014, and 2019 are selected as three key time points of this research.

### 2.2. The Interaction between UDI and ECC

**Figure 2:**
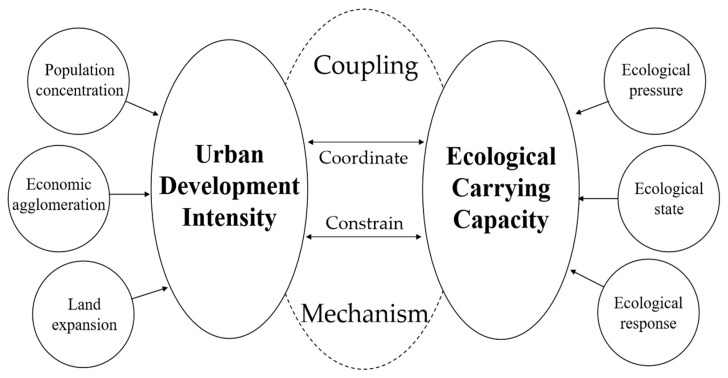
Coupling mechanism between UDI and ECC. Source: Summarized according to the literature [10,12,43].

Based on previous studies [10,12,43], urban development and the environment are mutually constrained and coordinated in a long time series. Therefore, the relationship between UDI and ECC can be defined as a coupling coordination relationship. That is to say, there is a dynamic equilibrium between the UDI and ECC. Under the impact of UDI, the ECC shows a law of resilience and constantly converts from a balance to a new balance for a certain period of time. We summarize the coupling mechanism between UDI and ECC and represent it in Figure 2. The specific explanation is as follows.

For instance, if the UDI continues to increase without regulation, the environmental pressure would be exacerbated, especially for ecologically fragile areas [50,51]. With the development of human society, human residences have moved from the countryside to the city, the population has grown excessively, and the urban space has expanded. Following this, the construction area of the city gradually expanded, and more and more skyscrapers have sprung up. These activities resulted in the erosion of ecological spaces and the continuous reduction of ecological resources such as green space and cultivated land. The ecological security of the whole area will be under serious threat. As a result, the ECC would inevitably decrease, and the initial equilibrium state would become imbalanced.

On the other hand, urban development is inseparable from the rigid constraints of the environment [12,43]. The construction and expansion of a city, the growth of the population, and the thriving of the economy inevitably consume many types of environmental resources: land resources, water resources, forest resources, etc. When the consumption of various resources exceeds the carrying capacity, it will affect the development of agriculture, industry, and even tertiary industry. What is worse, in extreme cases, it can lead to terrible natural disasters, such as soil erosion and drought. In that situation, the process of urban development has to slow down, or even stop. Then, the urban development intensity decreases.

In addition, a coupling coordination degree (CCD) model could well reflect the mutual influence and coordinated development of the two systems [52,53], which has been widely used to analyze the coupling coordination relationship between urban development and the environment [44,45,46,47]. It is also suitable for analyzing the relationship between UDI and ECC.

### 2.3. Research Framework

We took 22 districts and counties in the Three Gorges Reservoir Area (Chongqing section) as representatives, using the data from 2010, 2014, and 2019 to explore the interaction between UDI and ECC in ecologically fragile areas.

First, we comprehensively evaluated UDI from the aspects of population, economy, and land use in 2010, 2014, and 2019. We used a PSR model to assess the ECC in 2010, 2014, and 2019. In addition, the space–time characteristics of UDI and ECC were represented, which is useful for discussing the potential relationship between them.

Secondly, based on the understanding of the relationship of UDI and ECC, we used a coupling coordination degree (CCD) model to analyze the coupling coordination relationship between UDI and ECC in ecologically fragile areas, and the coupling degree of UDI and ECC (UDI–ECC coupling degree) and the coordination degree of UDI and ECC (UDI–ECC coordination degree) were obtained.

Then, we further explored the specific impact of the UDI on the coordinated development of UDI and ECC. The geographic weighted regression (GWR) model was adopted, which could embed its geographical coordinates into the model during a regression analysis and form a regression coefficient related to the location [54,55]. Thus, it could well reveal the impact of UDI on the UDI–ECC coordination in the three dimensions of population, economy, and land use.

**Figure 3:**
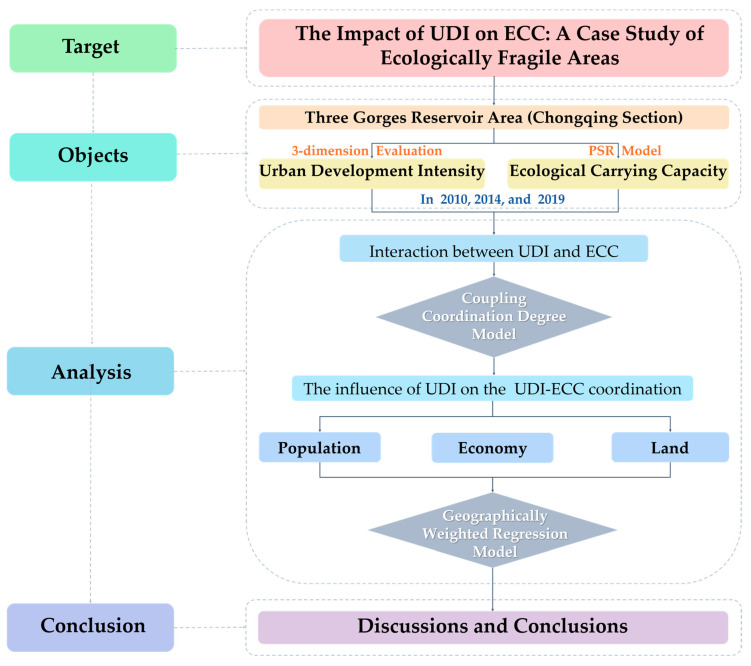
Research framework. Sources: Established by the authors.

Finally, according to the analysis results, we provide a discussion and give our conclusions. The research framework is shown in Figure 3.

### 2.4. Methods

#### 2.4.1. Evaluations of UDI and ECC

##### Evaluation of UDI

UDI reflects the compound state of urban land use, population, and economic development in a specific period [18,19,20]. Considering previous studies on the measurements of UDI [18,19,20] and the realistic situation in this paper, the comprehensive evaluation index system of UDI constructed included three first-grade indicators (population concentration intensity, economic agglomeration intensity, and land-use intensity). In addition, two second-grade indicators were selected under the first-grade indicators. The final index system is illustrated in Table 1, including six indicators.

Based on the multifactor comprehensive evaluation method, we also constructed a measurement model of UDI as follows:

(1) $$f(x_{i})=\sum_{i=1}^{n}x_{i}\cdot W_{j}$$

where n is the number of research units; f(x_i_) is the UDI value of the i-th unit; x_i_ is the standardized value of the i-th unit on the j-th index; and W_j_ is the index weight of the j-th index.

Referring to previous studies [18,19,20] and the real situation of the case, the calculation results of UDI were classified with the Jenk’s Natural Breaks Classification method, as shown in Table 2.

##### Evaluation of ECC

**Figure 4:**
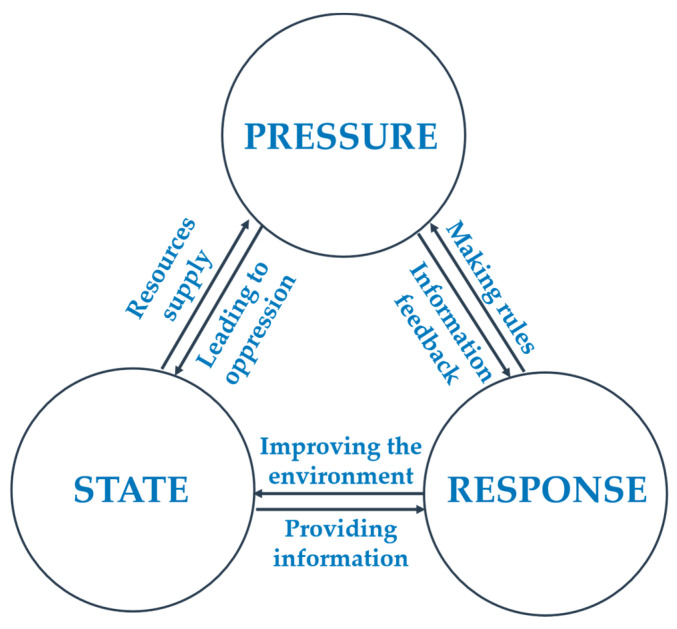
The framework of the PSR model. Source: Summarized by the authors according to the literature [35,36,37].

The ECC is the comprehensive capacity of the social–economic–natural complex ecosystem [30,31,32], including support capacity, supply capacity, and coordination capacity. The pressure-state-response (PSR) model can well reflect the dynamic changes of this comprehensive capacity in a certain period of time, which is helpful for assessing ecologically fragile areas. Therefore, we used the PSR model (Figure 4) to establish a comprehensive evaluation method for ECC.

The PSR model is composed of three parts [35,36,37]. The first part is the pressure level, i.e., the impacts on the environment caused by the activities that exert some pressure on the system. The second part is the state level, i.e., the description of the current situation of the system. The last one is the response level, which refers to the response to the system in the form of laws, regulations, and standards or environmental management behavior.

Based on the framework of the PSR model and previous studies [35,36,37] on the measurements of ECC, four indicators were selected to represent the pressure level, four to represent the state level, and three to represent the response level. The final index system is shown in Table 1, including 11 indicators.

At the same time, based on the multifactor comprehensive evaluation method, a measurement model of ECC was constructed. The details are as follows:

(2) $$g(y_{i})=\sum_{i=1}^{n}y_{i}\cdot W_{j}$$

where g(y_i_) is the ECC value of the i-th unit; y_i_ is the standardized value of the i-th unit on the j-th index; and W_j_ is the index weight of the j-th index.

Referring to the previous studies [35,36,37] and the realistic situation of the case, the calculation results of ECC are classified with Jenk’s Natural Breaks Classification method, as in the following Table 3.

##### Index Weight and Direction

The entropy method and the analytic hierarchy process (AHP) were used to calculate the weight of each index, which could achieve a good balance between subjective and objective [36,52]. The calculation formula is as follows:

(3) $$W_{j}=aW_{m}+(1-a)W_{n},$$

where n is the number of research units; W_j_ is the combination weight; W_m_ is the weight given by the AHP; W_n_ is the weight given by the entropy method; a is the proportion of the AHP weight in combination weight, with a value of 0.5; and 1 − a is the proportion of the entropy method in combination weight.

The positives and negatives of all the indicators were determined according to whether their roles are beneficial or harmful in the system in reality, as shown in Table 3.

#### 2.4.2. Coupling Coordination Degree Model

The coupling coordination degree model can well analyze the coupling coordination relationship between UDI and ECC [44,45,46,47]. It consists of a coupling degree model, a comprehensive evaluation index, and a coordination degree model.

##### Coupling Degree

Coupling degree can measure the interaction degree between the system or elements. To measure the coupling degree of UDI and ECC quantitatively, according to the coupling coordination model, the measurement model of the UDI–ECC coupling degree is constructed as follows:

(4) $$C=2{\left\{f(x)\times g(y)/{\left(f(x)+g(y)\right)}^{2}\right\}}^{1/2},$$

where C is the coupling degree, f(x) and g(y) are the index system function of UDI and the index system function of ECC, respectively.

##### Comprehensive Evaluation Index

The comprehensive evaluation index indicates the comprehensive development level of UDI and ECC. The function is as follows,

(5) T=af(x)+bg(y)

where a and b respectively represent the weight of UDI and ECC. According to previous studies [44,45,46,47] and comprehensive consideration, this paper considers that they have the same contribution, so a = b = 0.5 in the formula.

##### Coordination Degree

Coordination degree was constructed from the comprehensive evaluation index T and the coupling degree C, which can well measure the coordinated development level of the whole system [44,45,46,47]. The function is as follows:

(6) $$D=\sqrt{C•T},$$

where D is the coordination degree. Moreover, C ∈ [0,1], D ∈ [0,1], the coupling degree is directly proportional to the C value, and the coordination degree is directly proportional to the D value. When C = 1, the UDI and ECC reach the optimal coupling state. When D = 1, UDI and ECC reach the optimal coordination state.

At the same time, referring to the existing research results, the calculation results of the coupling degree and coordination degree were classified by the Equal-interval Classification method [44,45,46,47], as in Table 4.

#### 2.4.3. Moran’s I

Testing the spatial autocorrelation of dependent variables is a prerequisite for the application of the geographically weighted regression model [54,55]. We used the global Moran’s I to test whether there was a significant spatial correlation between the coordination degree of UDI and ECC. The specific calculation formula is as follows:

(7) $$I=\frac{n\sum_{i=1}^{n}\sum_{j=1}^{n}W_{ij}(x_{i}-\bar{x})(x_{j}-\bar{x})}{(\sum_{i=1}^{n}\sum_{j=1}^{n}W_{ij})\sum_{i=1}^{n}{\left(x_{i}-\bar{x}\right)}^{2}}$$

where n is the number of research units; x_i_ and x_j_ are the observed value of the marked i and j of research units; and W_ij_ is the spatial weight matrix. If the research units are adjacent, the result is 1; if they are not adjacent, the result is 0.

When I > 0, it means that the results are positively correlated, which represents that the research units belong to the aggregation spatial layout, and there is an autocorrelation. When I < 0, it means that the results are negatively correlated, which indicates that the research units and the research units belong to a decentralized spatial layout.

The global Moran’s I exponential statistics are generally tested by constructing value statistics (The Z value is the deviation between the attribute of element i and its average value); the calculation formula is as follows:

(8) $$Z=\frac{I-E(I)}{\sqrt{\mathrm{var}(I)}},$$

where E(I) is the expected value, var(I) is the variance, and the evaluation standard of the Z value is as in Table 5.

#### 2.4.4. Geographically Weighted Regression Model

The geographically weighted regression (GWR) model is a spatial econometric regression analysis method [54,55]. It can reflect different influences of a variable in the region based on different geographical coordinates.

Therefore, this paper analyzes the spatiotemporal differentiation characteristics of the impact of UDI on the UDI–ECC coordination by the GWR model. The function of the model is presented as follows:

(9) $$y_{i}=a_{i}{}_{o}(u_{i},v_{i})+\sum_{i=1}^{n}a_{i}{}_{k}(u_{i},v_{i})\cdot x_{i}{}_{k}+\varepsilon_{i}$$

where the coordinate of the i-th point is (u_i_,v_i_); x_ik_ is the independent variable of the ith point; a_io_(u_i_,v_i_) is the estimated value of the constant term of the i-th point, a_ik_(u_i_,v_i_) is the estimated value of the regression parameter of the k-th independent variable at the i-th point; n is the number of regression terms, and ε_i_ is the residual correction term.

To verify the fitting effect of the GWR model in this paper, the fitting parameters of the GWR model will be compared with the traditional least square linear regression model. The ordinary least squares (OLS) model is a global linear regression model, which uses the best fitting line method to analyze the relationship between explained variables and explanatory variables [55]. The calculation formula is as follows:

(10) $$y_{i}=\beta_{o}+\sum_{k=1}^{n}\beta_{k}x_{ik}+\varepsilon_{i},$$

where y_i_ is the value of the dependent variable at i-th point; x_ik_ is the value of the k-th independent variable at the i-th point; k is the number of independent variables; β_o_ is the constant term; β_k_ is the regression coefficient of the k-th independent variable; n is the number of regression terms, ε_i_ is the residual.

In this paper, the weight function of geographically weighted regression is calibrated by the adaptive method, and the minimum Akaike information criterion (AIC) method [54,55] is used to determine the bandwidth. The Akaike information criterion (AIC) is a technique that measures the goodness of an estimated statistical model [56]. As for the calculation results, the corrected Akaike information criterion (*AICc*) can help to compare different regression models. *R*^2^ (the goodness of fit) [57] indicates the degree of explanation of the regression equation to the changes of dependent variables. Adjusted *R*^2^ is the calculation result of variable compensation based on *R*^2^, which could minimize the calculation error [57].

Both geographically weighted regression analysis and least square linear regression analysis were completed in ArcGIS 10.2 (ESRI, Redlands, CA, USA).

### 2.5. Data

#### 2.5.1. Data Resources

The social and economic data mainly came from the Statistical Yearbook published by the Chongqing Municipality or the governments of each district and county (in the Three Gorges Reservoir) from 2010 to 2020 (the statistical yearbook for a certain year only contains data up to the previous year, which was decided by the Statistics Department in China). The environmental data mainly came from the Environmental Statistical Bulletin of Chongqing and the Water Resources Statistical Bulletin of Chongqing from 2010 to 2019. The spatial data mainly came from the geospatial cloud data of Chongqing from 2010 to 2019. The missing data were obtained by interpolation using the nearest year’s data, to ensure the authenticity and integrity of the data.

#### 2.5.2. Standardization Treatment

Due to the differences in dimensions, meanings, and attributes of the original data, it was necessary to eliminate the influence caused by the differences. Thus, we used the Z-score Equation (10) to standardize the original data, as follows:

(11) $$x_{i}^{\prime }=\frac{x_{i}-\bar{x}}{\sqrt{\sum_{i-1}^{n}{\left(x_{i}-\bar{x}\right)}^{2}/\left(n-1\right)}}$$

where x_i_ is the original index value and $x_{i}^{\prime }$ is the standardized value of x_i_.

## 3. Results

### 3.1. Spatiotemporal Variations of UDI

Based on the evaluation index system of UDI, we obtained the UDI values of the 22 districts and counties in the Three Gorges Reservoir Area (Chongqing section) for the years 2010, 2014, and 2019. Then, we analyzed their spatial distributions and temporal changes by ArcGIS 10.2 (ESRI, Redlands, CA, USA) and SPSS 25 (IBM, Armonk, NY, USA).

**Figure 5:**
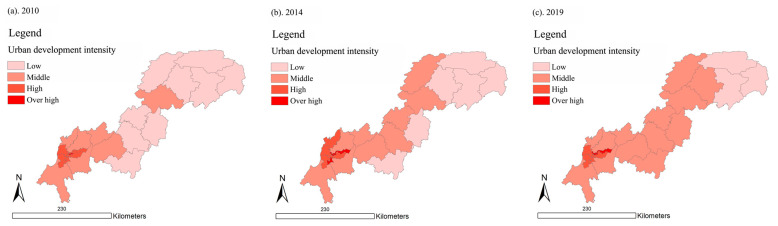
Spatial distribution of UDI in the Three Gorges Reservoir Area (Chongqing section) in year 2010, 2014, and 2019 (presented in subfigure **a**–**c**, respectively). Sources: Calculation results of UDI in the Three Gorges Reservoir Area (Chongqing section).

As shown in Figure 5, in 2010, 2014, and 2019, the cities with relatively high UDI were mostly distributed in the upper reaches of the Three Gorges Reservoir Area, close to the main urban area of Chongqing. On the contrary, the cities with relatively low UDI were mostly located in the lower reaches of the Three Gorges Reservoir Area. Yuzhong District had the highest UDI value, and the average of the UDI over the three years reached 7.82, which means it stayed at an over high level. Wuxi County had the lowest UDI value, and the UDI average in the three years was as low as 0.147, which means it stayed at a low level. This distribution phenomenon may be affected by the urban development pattern of Chongqing. In particular, most of the central urban areas of Chongqing were located in the upper reaches of the Three Gorges Reservoir Area, the UDI values of which were relatively high.

**Figure 6:**
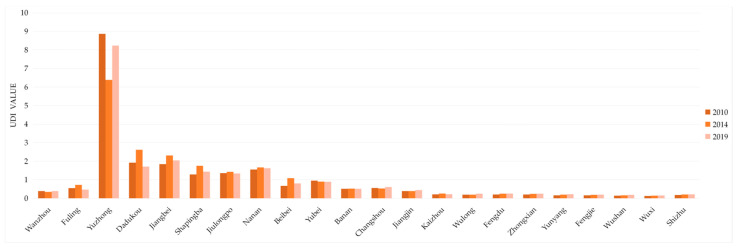
Changes of UDI in the Three Gorges Reservoir Area (Chongqing section). Sources: Calculation results of UDI in the Three Gorges Reservoir Area (Chongqing section).

As shown in Figure 6, the variations of UDI average values of the counties were not significant in 2010, 2014, and 2019, which indicates that the UDI in the Three Gorges Reservoir Area was nearly constant on the whole. However, the UDI variance of districts and counties in the Three Gorges Reservoir Area (Chongqing section) was about 3.4 in 2010 and 2.92 in 2019, a decline of 0.48. That is, the UDI differences between districts and counties narrowed over time. This indicates that the western development projects in the western region of China have indeed achieved corresponding results for the past 10 years.

On the whole, the spatial distribution of UDI in the Three Gorges Reservoir Area (Chongqing section) is high upstream and low downstream. In the terms of time, the differences between districts and counties are shrinking, and a balanced development trend between districts and counties is appearing.

### 3.2. Spatiotemporal Variations of ECC

After calculation, the ECC values in 2010, 2014, and 2019 were obtained. Then, the spatial distributions and temporal variations were analyzed by ArcGIS 10.2 and SPSS 25.

**Figure 7:**
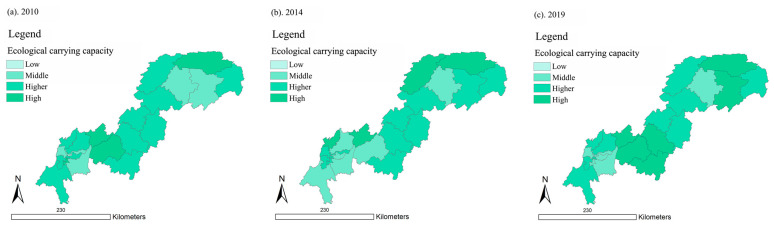
Spatial distribution of ECC in the Three Gorges Reservoir Area (Chongqing section) in year 2010, 2014, and 2019 (presented in subfigure **a**–**c**, respectively). Sources: Calculation results of ECC in the Three Gorges Reservoir Area (Chongqing section).

As shown in Figure 7, the results show that the areas with relatively high ECC in the Three Gorges Reservoir Area (Chongqing section) were mostly distributed in the middle--lower reaches. In 2010, 2014, and 2019, Wuxi County had the highest ECC value with an average value of 1.77, which means it stayed at a high level. Yuzhong District had the lowest ECC value with an average value of 0.39, which means it stayed at a low level. This situation was opposite to the distribution of UDI.

**Figure 8:**
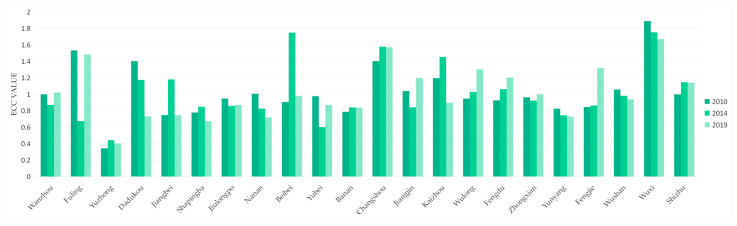
Changes of ECC in Three Gorges Reservoir Area (Chongqing section). Sources: Calculation results of ECC in Three Gorges Reservoir Area (Chongqing section).

As shown in Figure 8, the average ECC values of the Three Gorges Reservoir Area (Chongqing section) in 2010, 2014, and 2019 were all about 1, which means almost the whole area was in the higher level for ECC. The variance values in 2010, 2014, and 2019 all stayed at about 0.1, indicating that the distributions of ECC between districts and counties were relatively concentrated and stable.

In summary, the ECC of the Three Gorges Reservoir Area (Chongqing section) formed a spatial pattern of lower upstream and higher downstream. In terms of time, the ECC stayed at a high level, and the changes of ECC were not significant. At the same time, the high UDI areas had low ECC, while the low UDI areas had high ECC.

### 3.3. Coupling Coordination Degree between UDI and ECC

In 2010, 2014, and 2019, the UDI–ECC coupling degree in the Three Gorges Reservoir Area (Chongqing section) remained at a medium or low level.

**Figure 9:**
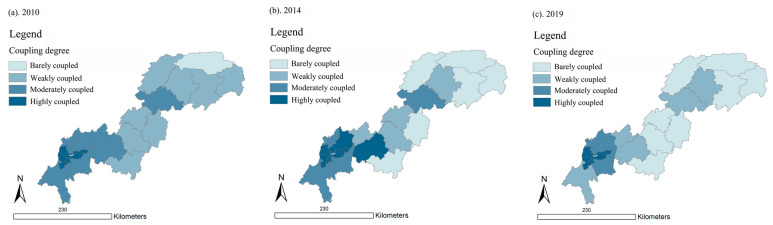
Spatial distribution of coupling degree in the Three Gorges Reservoir Area (Chongqing section) in year 2010, 2014, and 2019 (presented in subfigure **a**–**c**, respectively). Sources: Calculation results of the CCD model.

As shown in Figure 9, Wuxi County and Yuzhong District had the lowest UDI–ECC coupling degree, showing that they were barely coupled. The districts in the high coupling stage were Nan’an District, Dadukou District, Shapingba District, Jiangbei District, and Jiulongpo District. Their UDI–ECC coupling degree values were all over 0.8 in 2010, 2014, and 2019. Meanwhile, most of them were distributed in the upper reaches, close to the main area of Chongqing.

The average values of the UDI–ECC coupling degree in 2010, 2014, and 2019 were 0.566, 0.576, and 0.502, respectively. We can see that the average value declined by 0.064 from 2010 to 2019. This may indicate that the overall UDI–ECC coupling degree of the Three Gorges Reservoir Area (Chongqing section) had a slight downward trend.

**Figure 10:**
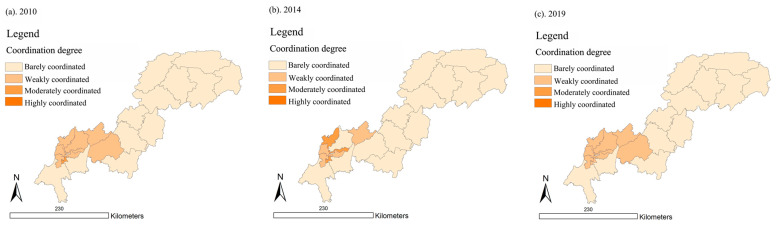
Spatial distribution of coordination degree in the Three Gorges Reservoir Area (Chongqing section) in year 2010, 2014, and 2019 (presented in subfigure **a**–**c**, respectively). Sources: Calculation results of the CCD model.

As shown in Figure 10, in 2010, 2014, and 2019, the UDI–ECC coordination degrees of the Three Gorges Reservoir Area (Chongqing section) were mostly at a low level. Specifically, there were 13, 15, and 13 districts and counties that had a UDI–ECC coordination degree values lower than 0.4, in 2010, 2014, and 2019, respectively. Meanwhile, most of the districts and counties with relatively low coordination were distributed in the lower reaches, and parts of them were distributed in the middle and upper reaches.

On the whole, the average values of UDI–ECC coordination were 0.377, 0.367, and 0.338 in 2010, 2014, and 2019, respectively, showing a decreasing trend over time. This demonstrates that the UDI–ECC coordination degree value declined over the past 10 years, and most of the districts and counties in the Three Gorges Reservoir Area (Chongqing section) were in a barely coordinated state.

**Figure 11:**
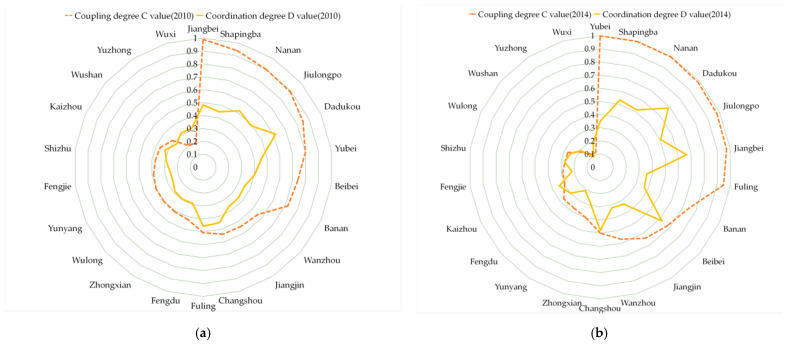

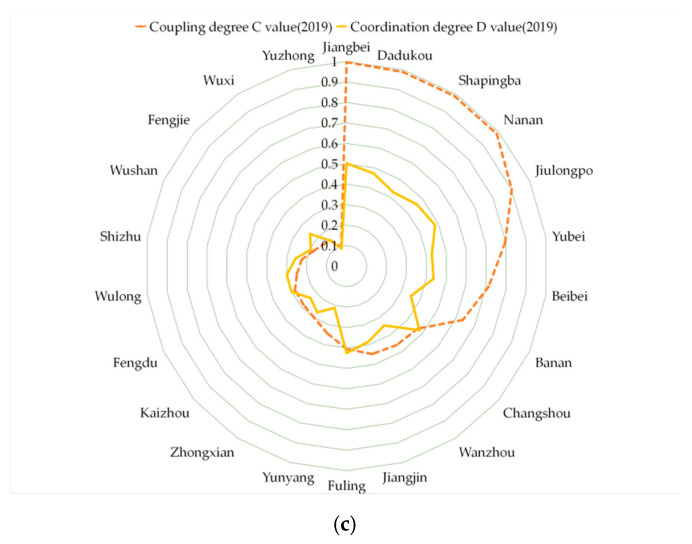
Comparison of UDI-ECC coupling degree and UDI–ECC coordination degree in 2010 (**a**), 2014 (**b**), and 2019 (**c**). Sources: Calculation results of the CCD model.

As we can see from Figure 11, it is worth noting that the UDI–ECC coupling degrees and UDI–ECC coordination degrees of Yuzhong District (high UDI, low ECC), Wuxi County (low ECC, high ECC), and Wushan County (low ECC, high ECC) were all at a relatively low level in 2010, 2014, and 2019. This indicates that the UDI–ECC coupling degrees and UDI–ECC coordination degrees of these areas were barely coupled and weakly coordinated for a long time.

Therefore, the values of UDI and ECC should be kept in a relatively balanced range, which is more helpful for the coordination of UDI and ECC.

### 3.4. The Impacts of UDI on UDI–ECC Coordination Degree

According to the relevant research [11,18,20], the coordination degree value in the CCD model can well measure the coordinated development level of the whole system. Therefore, the UDI–ECC coordination degree can be used to represent the coordinated development levels between UDI and ECC.

After a comparison with the previous results in Section 3.1 and Section 3.3, we found that the coordination degree of the regions with higher UDI is also higher. It is worth exploring what kind of impact UDI has on the UDI–ECC coordination degree.

To figure out the answers, this section will use a GWR model to analyze the internal influence of the UDI on the UDI–ECC coordination degree in different dimensions.

#### 3.4.1. Spatial Autocorrelation Test

Based on the evaluation criteria in Table 5, the global Moran’s I values of the UDI–ECC coordination degree were greater than 0 in 2010, 2014, and 2019, indicating that the spatial distribution of the UDI–ECC coordination had a positive spatial correlation. As shown in Table 6, in 2010, 2014, and 2019, the Z values were 5.225, 3.282, and 3.708, respectively, and higher than the test value of 2.58. In other words, they were all in the 99% confidence interval of normal distribution. There was indeed a significant spatial correlation between the coordination degree of UDI and ECC.

#### 3.4.2. Selection of Independent Variables and Model

For the independent variables, we first selected six indicators from the three dimensions of population, economy, and land: urbanization rate, population density, economic density, per capita GDP, construction land proportion, and per capita construction land area. After the significance test and multicollinearity test, only the indicators population density, per capita GDP, and per capita construction land area were chosen to represent the UDI from the dimensions of population, economy, and land, respectively.

Motivated by this, we used the GWR model and OLS model to analyze the impacts of UDI on UDI–ECC coordination in 22 districts and counties in 2010, 2014, and 2019, respectively. The calculation results of the GWR model and the OLS model are shown in Table 7. By comparison, we found that *R*^2^ (the goodness of fit) of the GWR model were higher than that of the OLS model, and *AICc* were lower than that of the OLS model.

Furthermore, we found that the fitting parameters of the OLS model and GWR model were almost unchanged with per capita construction land area as the independent variable. However, the fitting parameters of the two models changed significantly when population density or per capita GDP was an independent variable. This indicates that the impacts of UDI on UDI–ECC coordination under the land-use dimension may not have spatial differences, and the spatial differences were more reflected in the impacts of population and economy.

Therefore, in this study, the GWR model has a better fitting effect and a stronger explanation of geographic differences than the OLS model. It is appropriate to choose the GWR model to explain the impacts of UDI on UDI–ECC coordination.

#### 3.4.3. Impacts of UDI on UDI–ECC Coordination Degree

The impacts of UDI on the UDI–ECC coordination degree in different dimensions are analyzed by the GWR model in this section.

For the UDI in population dimension (P-UDI), the average regression coefficients in 2010, 2014, and 2019 were 0.1214, 0.1667, and 0.1644, respectively, which indicates that the impact of P-UDI on the UDI-ECC coordination was gradually strengthened in the Three Gorges Reservoir Area (Chongqing section).

**Figure 12:**
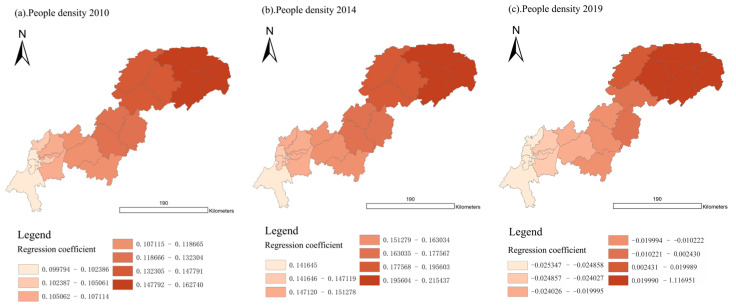
Spatial distribution of regression coefficient of P-UDI in year 2010, 2014, and 2019 (presented in subfigure **a**–**c**, respectively). Sources: Calculation results of the GWR model from the population dimension.

As shown in Figure 12, in 2010 and 2014, the regression coefficients in 22 districts and counties were all positive, and the values in the upstream area were lower than in the downstream area. However, in 2019, the impact of P-UDI on the UDI-ECC coordination appeared an obvious spatial differentiation. In the middle and upper reaches of the Three Gorges Reservoir Area (Chongqing section), there were weak/negative values, while in the lower reaches, there were strong/positive values.

**Figure 13:**
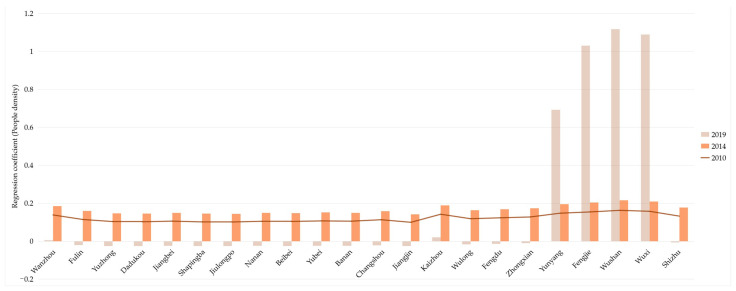
Changes of regression coefficient of P-UDI in the Three Gorges Reservoir Area (Chongqing section). Sources: Calculation results of the GWR model from the population dimension.

Specifically, as Figure 13 shows, in 2019, the P-UDI regression coefficients in sixteen districts and counties changed from positive to negative, and only six remained positive. In particular, the values of four districts and counties in Northeast Chongqing, Yunyang, Fengjie, Wushan, and Wuxi, increased significantly from 2014 to 2019. This shows that the impact of P-UDI on the UDI-ECC coordination has changed from a positive effect to a negative effect in most areas.

Therefore, for good coordination in the Three Gorges Reservoir Area (Chongqing section), those areas with negative values should appropriately control the excessive population aggregation, while those with positive values should appropriately guide the population growth.

For UDI in the economic dimension (E-UDI), the average regression coefficients of 2010, 2014, and 2019 were 0.0943, 0.1536, and 0.045, respectively, indicating that the E-UDI on the UDI–ECC coordination first strengthened and later weakened over the past 10 years.

**Figure 14:**
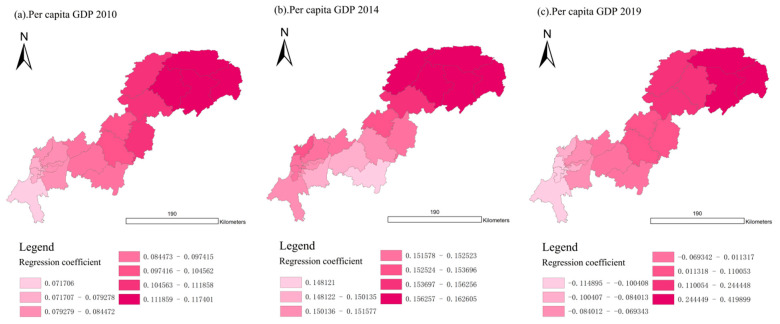
Spatial distribution of regression coefficient of E-UDI in year 2010, 2014, and 2019 (presented in subfigure **a**–**c**, respectively). Sources: Calculation results of the GWR model from the economic dimension.

As shown in Figure 14, similar to the population dimension, in 2010 and 2014, the regression coefficients of 22 districts and counties in the Three Gorges Reservoir Area (Chongqing section) were positive, and the upper reaches were lower than the lower reaches. When it came to 2019, the impact of E-UDI on the UDI–ECC coordination showed an obvious spatial differentiation, i.e., the values were negative in the upper reaches and positive in the middle and lower reaches.

**Figure 15:**
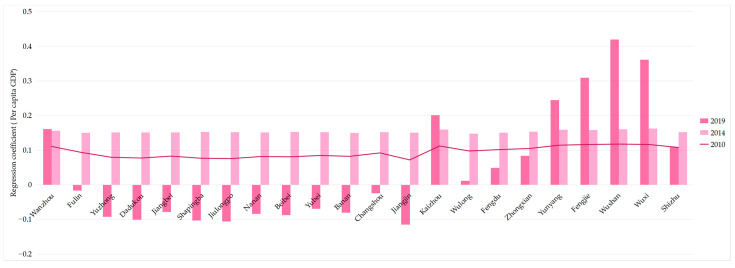
Changes of regression coefficient of E-UDI in the Three Gorges Reservoir Area (Chongqing section). Sources: Calculation results of the GWR model from the economic dimension.

Specifically, as Figure 15 shows, in 2019, the E-UDI regression coefficients of twelve districts and counties changed from positive to negative, and only ten remained positive. In particular, the values of five districts and counties located in the middle and lower reaches, including Kaizhou, Yunyang, Fengjie, Wushan, and Wuxi, have increased significantly during 2014–2019. This shows that the impact of E-UDI on the UDI–ECC coordination has changed from positive effect to negative effect in more than half of the regions in 2019.

Therefore, we should pay attention to ensuring a balanced economic development both in the upstream and downstream areas of the Three Gorges Reservoir Area (Chongqing section), and increasing investment in underdeveloped areas, so as to improve the coordination development for the whole region.

For UDI in the land-use dimension (L-UDI), the average regression coefficients of L-UDI in 2010, 2014, and 2019 were 0.1255, 0.2042, and 0.1685, respectively, indicating that the impact of L-UDI on UDI–ECC coordination degree was significantly enhanced from 2010 to 2014. From 2014 to 2019, urbanization in the Three Gorges Reservoir Area (Chongqing section) entered the transition period, and the growth of UDI slowed down. Therefore, the regression coefficients of L-UDI in 2019 were lower than those in 2014. To summarize, L-UDI has a low positive correlation with UDI–ECC coordination.

**Figure 16:**
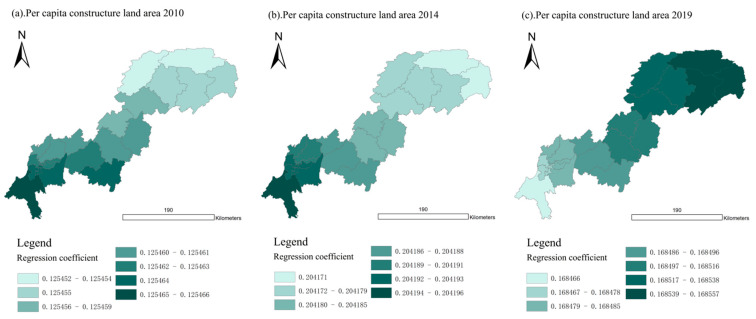
Spatial distribution of regression coefficient of L-UDI in year 2010, 2014, and 2019 (presented in subfigure **a**–**c**, respectively). Sources: Calculation results of the GWR model from the land-use dimension.

**Figure 17:**
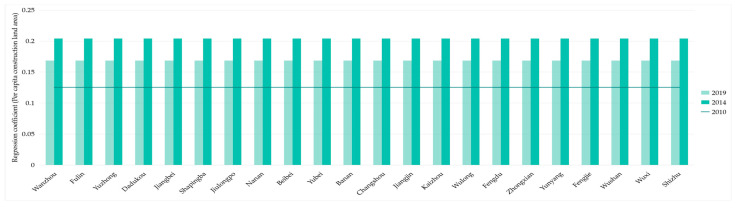
Changes of regression coefficient of L-UDI in the Three Gorges Reservoir Area (Chongqing section). Sources: Calculation results of the GWR model from the land-use dimension.

As shown in Figure 16, in 2010, 2014, and 2019, the regression coefficients of 22 districts and counties in the Three Gorges Reservoir area were all positive, and the values of regression coefficients were nearly close. Furthermore, as shown in Figure 17, in 2010, 2014, and 2019, the variance of the L-UDI regression coefficient of the whole region remained around 0. Therefore, there was no spatial differentiation. These were consistent with our inference in Section 3.4.2.

In addition, the regression coefficient of L-UDI has been kept at a low positive value in the past 10 years, which indicates that the appropriate improvement of L-UDI will be conducive to UDI–ECC coordination.

By comparing the mean values of the regression coefficient of UDI in different dimensions, we can find that the mean value of the L-UDI regression coefficient (0.166) > the mean value of the P-UDI regression coefficient (0.1508) > the mean value of E-UDI regression coefficient (0.0977). This shows that the UDI–ECC coordination degree was affected more by L-UDI and P-UDI than by E-UDI. What is more, the impacts of the dimensions on the UDI–ECC coordination degree all showed a low degree of positive correlation, which means that an appropriate increase in UDI may be helpful for the improvement of the UDI–ECC coordination degree.

## 4. Discussion

The fundamental spatial distribution pattern of ECC is determined by the natural environment [28,58]. The terrain is high in the east and low in the west in the Three Gorges Reservoir Area (Chongqing section) [38]. The area with steep terrain has more abundant natural resources, such as forest, water, etc. In this study, a stable spatial distribution with relatively low ECC upstream and relatively high ECC downstream reflects this point. The spatial distribution of ECC is also affected by social and economic factors [30,32]. In 2008, the National Council released the National Ecological Function Zoning, identifying the Three Gorges Reservoir Area as one of 25 key ecological function areas [42]. Then, in 2013, the Chongqing municipal government issued the Ecological Conservation Function Zone Planning, mentioning the important role of the Three Gorges Reservoir area (Chongqing section) in maintaining the overall ecological quality [42]. The varying of ECC in the Three Gorges Reservoir Area (Chongqing section) over the past 10 years is not significant, and the whole ECC stays at a high level. Therefore, we can see that these policies indeed played a positive role, and we should pay more attention to the implementation of relevant policies in the future.

Previous studies suggested that effective land use is of positive significance in terms of ecosystem services and ECC [7]. The results of this study demonstrate the same points. In the Three Gorges Reservoir Area (Chongqing section), the UDI values were relatively high upstream and relatively low downstream in 2010, 2014, and 2019. With the release of a series of policies [42], such as the Chengdu Chongqing Urban Agglomeration Planning in 2016, etc., the districts and countries in the upper reaches have had more economic investment and opportunities, and the UDI has developed faster than in the lower reaches. Meanwhile, the ECC has maintained a high level over the past 10 years, which indicates that reasonable guidance of UDI is conducive to maintaining a high ECC. However, it is worth noting that areas with extremely high UDI are linked to extremely low ECC. Therefore, we speculate that the UDI and ECC may be mutually restricted within a certain range, but this situation is not obvious outside the range.

Based on a further analysis of the relationship between UDI and ECC, we found that the UDI–ECC coupling degree and UDI–ECC coordination degree of the Three Gorges Reservoir Area (Chongqing section) were mostly in the state of moderate coupling and weak coordination in 2010, 2014, and 2019. Previous studies [12] have found that the coupling coordination relationship in most regions of China was at a low level. Therefore, the results of this study also verify this point, indicating that there are some contradictions between urban development and the environment in many parts of China, especially in ecologically fragile areas.

Previous studies [10,45,46] have shown that a higher urban development level, such as in the Yangtze River Delta and Pearl River Delta, is linked to a higher coordination degree between urban development and the environment. However, the specific impact of UDI on the coordination degree between urban development and the environment requires further analysis. In this study, we found that the areas with relatively high UDI correlate to the higher UDI–ECC coordination degree—that is, also the area close to the main urban area of Chongqing. At the same time, the UDI–ECC coordination degree often stayed at a low level in areas where the values of UDI and ECC were extreme (too high or too low). This verifies our initial assumption that too high a UDI value will restrict the ECC. Therefore, we believe that the UDI level can promote the coordinated development between UDI and ECC to a certain extent. The values of UDI and ECC in the region should be kept to a relatively narrow range, not too high or too low, for the coordinated development of urban spaces and the environment.

Moreover, we have analyzed the specific impact of UDI on UDI–ECC coordination by the GWR model. The UDI of different dimensions and UDI–ECC coordination degree all had a weakly positive correlation in 2010, 2014, and 2019. In particular, the UDI changes in population and land had a greater positive impact on UDI–ECC coordination than changes in the economy. This means that the reasonable improvement of UDI was, to a certain extent, conducive to the UDI–ECC coordination. This confirms our hypothesis about the impact of UDI on UDI–ECC coordination, and applies to most areas in the Three Gorges Reservoir Area (Chongqing section). Motivated by this, we also found that the impacts of P-UDI and E-UDI on the UDI–ECC coordination degree had obvious spatial differentiations, while the impacts of L-UDI on the UDI–ECC coordination degree had no spatial differentiation yet showed a slightly positive correlation in the whole region. Therefore, we put forward some corresponding suggestions for improvement based on the specific research results, as follows.

Specifically, in the regions with relatively high UDI, i.e., the upper reaches areas of the river, the impacts of P-UDI on the UDI–ECC coordination degrees went from positive to negative over time. This shows that the improvement in P-UDI used to have a positive effect on the degree of coordination in a certain range, but now has a moderately negative effect. The impacts of E-UDI on UDI–ECC coordination showed a similar development trajectory, and were relatively lower than those of P-UDI. Therefore, in areas with high UDI, more attention should be paid to controlling overpopulation and encouraging a balanced economic distribution, which will be useful for the coordinated development of urban spaces and the environment.

For regions with relatively low UDI, i.e., the lower reaches, the impacts of P-UDI and E-UDI on UDI–ECC coordination were positive and had a tendency to strengthen. In particular, the impacts of P-UDI were greater than those of E-UDI. This indicates that appropriate improvements in UDI in the population and economy will be conducive to UDI–ECC coordination in areas with low UDI. Therefore, we should improve the UDI in the region appropriately, such as by leading to population aggregation and enhancing the economic investments, to facilitate the coordinated development of urban spaces and the environment.

## 5. Conclusions

In this paper, taking the Three Gorges Reservoir Area (Chongqing section) as a typical ecologically fragile area, the UDI and ECC in this area in 2010, 2014, and 2019 were evaluated, and the internal relationship of UDI and ECC was analyzed using the CCD and GWR models. On this basis, the impact of UDI on the environment was discussed in depth.

Specifically, we found that: (1) The distributions of UDI and ECC are different. The UDI and ECC may be mutually restricted to some extent. (2) UDI and ECC are mostly moderately coupled and lowly coordinated. Extreme UDI and ECC values are linked to extreme coordination degrees. (3) The UDI–ECC coordination degree tends to be higher in areas with higher UDI. However, it is not suitable for a case with extreme values. (4) UDI can promote the coordinated development of UDI and ECC to some extent. (5) The UDI changes in population and land have a greater impact on UDI–ECC coordination than those in the economy. (6) For areas with different UDI, the measurements to promote the coordinated development of UDI and ECC should be different. Specifically, in areas with higher UDI, i.e., the upper reaches, more attention should be paid to controlling overpopulation and encouraging a balanced economy distribution, which will be conducive to the coordinated development of urban spaces and the environment. Meanwhile, in areas with lower UDI, i.e., the lower reaches, promotion of population aggregation and economic investment will encourage the coordinated development of urban spaces and the environment.

The results of this study have revealed the relationship between UDI and ECC and the specific impact of UDI on the coordinated development of the two, and enriched our theoretical understanding of the impact of urban development on the environment. This is helpful for city managers and policymakers in ecologically fragile areas to formulate different control measures for UDI under different dimensions and promote the realization of long-term sustainable development and a virtuous circle.

This paper has some limitations. The impacts of UDI in different dimensions on the coordinated development of UDI and ECC have been revealed. We also found that some areas in the Three Gorges Reservoir Area (Chongqing section) are facing extremely imbalanced coordinated degree values, if let be, which will be detrimental to the ecological security for the whole region. However, due to the limitations of the methods and the short time selected, the specific desirable range of UDI remains to be further studied, especially for the imbalanced areas. Therefore, we expect to conduct targeted research in imbalanced areas in the future, using system models such as neural network algorithms and system dynamics models to calculate the numerical rational range of UDI from a long time series. This will be useful for the dynamic monitoring of UDI, and potential ecological security problems can be prevented in time.

## Figures and Tables

**Table 1:** Evaluation index system of UDI and ECC.

Target Layer	Element Layer	Index Layer	Unit	Weight	Direction
Urban development intensity (UDI)	Population concentration intensity	Population density	Person/km^2^	0.2455	+
		Urbanization rate	%	0.1116	+
	Economic agglomeration intensity	Economic density	10,000 yuan/km^2^	0.1759	+
		Per capita GDP	Ten thousand yuan	0.1129	+
	Land-use intensity	Per capita construction land area	100 m^2^/person	0.1324	+
		Proportion of urban built-up area in total area	%	0.2217	+
Ecological carrying capacity (ECC)	Ecological pressure	Per capita industrial waste water discharge	T/person	0.1036	_
		Per capita industrial solid waste discharge	10,000 m^3^/person	0.0917	_
		Per capita industrial emission	T/person	0.0937	_
		Energy consumption per 10,000 yuan output value	T standard coal/person	0.1262	_
	Ecological state	Green coverage rate of built-up area	%	0.0656	+
		Forest coverage	%	0.0832	+
		Per capita cultivated land area	Hm^2^/person	0.0889	+
		Per capita water resources	10,000 m^3^/person	0.1666	+
	Ecological response	Standard rate of industrial waste water discharge	%	0.0581	+
		Synthesis utilization rate of industrial waste	%	0.0558	+
		Proportion of environmental protection investment in GDP	%	0.0666	+

**Source:** Chongqing Statistical Yearbook (the districts and counties data section) from 2011, 2015, and 2020 (the Statistical Yearbook for a certain year only contains the data up to the previous year, which was decided by the Statistics Department in China); Historical Remote Sensing Data of 22 districts and counties for 2010, 2014, and 2019; Environmental Statistics Bulletin and Water Resources Statistics Bulletin of Chongqing from 2010, 2014, and 2019. The missing data were obtained by interpolation method.

**Table 2:** Classification standard of UDI.

Low Level	Middle Level	High Level	Over High Level
<0.22	0.22~0.96	0.96~1.93	>1.93

Sources: Literature [18,19,20].

**Table 3:** Classification standard of ECC.

Low Level	Middle Level	Higher Level	High Level
<0.34	0.34~0.85	0.85~1.19	>1.19

Sources: Literature [35,36,37].

**Table 4:** Classification standard of coupling coordination degree.

Coupling Degree C Value	Coupling Degree	Coordination Degree D Value	Coordination Level
0.8 < C < 1	Highly coupled	0.8 < D ≤ 1	Highly coordinated
0.5 < C ≤ 0.8	Moderately coupled	0.6 < D ≤ 0.8	Moderately coordinated
0.3 < C ≤ 0.5	Weakly coupled	0.4 < D ≤ 0.6	Weakly coordinated
0 < C ≤ 0.3	Barely coupled	0 < D ≤ 0.4	Barely coordinated

Sources: Literature [44,45,46,47].

**Table 5:** Value evaluation criteria.

Z Value (Standard Deviation)	*p* Value (Probability)	Confidence
<−1.65 or >+1.65	<0.10	90.0000%
<−1.96 or >+1.96	<0.05	95.0000%
<−2.58 or >+2.58	<0.01	99.0000%

Sources: Literature [54,55].

**Table 6:** Moran’s I value of coordination in Three Gorges Reservoir Area (Chongqing section).

Year	Moran’s I	Z Value (Standard Deviation)	*p* Value (Probability)	Confidence
2010	0.506	5.225	0	99%
2014	0.304	3.282	0.001	99%
2019	0.347	3.708	0.0002	99%

Sources: Calculation results of the global Moran’s I.

**Table 7:** The fitting parameters of the GWR model and OLS model (2010, 2014, and 2019).

Year	Index	GWR Model			OLS Model		
		*AICc*	*R* ^2^	Adjusted *R*^2^	*AICc*	*R* ^2^	Adjusted *R*^2^
2010	Population density	−48.4281	0.5502	0.4978	23.4375	0.4638	0.4370
	Per capita GDP	−46.8069	0.5175	0.4594	33.9026	0.4481	0.4205
	Per capita construction land area	−82.6492	0.8951	0.8898	3.5383	0.8951	0.8898
2014	Population density	−21.2195	0.4136	0.3581	30.4726	0.3539	0.3216
	Per capita GDP	−21.5387	0.4222	0.3669	32.4790	0.3776	0.3465
	Per capita construction land area	−42.8567	0.7662	0.7544	18.4766	0.7662	0.7544
2019	Population density	−52.3502	0.81	0.7568	109.4140	0.1081	0.0636
	Per capita GDP	−40.9364	0.6403	0.5625	34.7369	0.0588	0.0117
	Per capita construction land area	−58.5241	0.7879	0.7773	11.8778	0.7879	0.7773

Sources: Calculation results of the GWR model. Note: A lower *AICc* value means the model better fits the observed data. The *R*^2^ value varies from 0 to 1. The larger the value, the better. The adjusted *R*^2^ is usually lower than the *R*^2^ value. Its evaluation standard is the same as for *R*^2^.

## Data Availability

Data available on request due to restrictions eg privacy or ethical. The data presented in this study are available on request from the corresponding author. The data are not publicly available due to [Part of the data is secondary processing data, involving a large amount of original data and so many types of data, which are not suitable for forming a unified database.].
